# Wait-and-See Approach or Gluten-Free Diet Administration—The Rational Management of Potential Coeliac Disease

**DOI:** 10.3390/nu13030947

**Published:** 2021-03-15

**Authors:** Anna Szaflarska-Popławska

**Affiliations:** Department of Pediatric Endoscopy and Gastrointestinal Function Testing, Ludwik Rydygier Collegium Medicum, Bydgoszcz, Nicolaus Copernicus University of Torun, Jagiellonska 13-15, 85-067 Bydgoszcz, Poland; aszaflarska@wp.pl; Tel.: +48-52-5854-888; Fax: +48-52-585-4799

**Keywords:** potential coeliac disease, gluten-free diet, management

## Abstract

Potential celiac disease (PCD) is a heterogeneous disease; only some patients develop full celiac disease (CD), characterised by advanced atrophic changes in the small intestine. Few accurate prognostic factors exist for the progression of PCD; therefore, therapeutic decisions should be made on an individual basis in each case. Patients with clinical gastroenterological or parenteral symptoms often benefit from a gluten-free diet, and those left on a diet containing gluten should receive clinical, serological and histopathological supervision.

## 1. Introduction

Celiac disease (CD) is a systemic disease characterized by the development of enteropathy, frequently accompanied by clinical symptoms of the gastrointestinal and/or non-gastrointestinal tract. Most CD patients demonstrate genetically predisposed sensitivity to the prolamines contained in wheat, rye and barley. For many years, the vast majority of patients, both those at risk of CD and those with existing symptoms, have been diagnosed based on the presence of CD-specific serum antibodies. Such serological testing has also commonly been used in population screening studies. In CD, the severity of damage to the small intestine can range from isolated intraepithelial lymphocytosis to complete atrophy of intestinal villi and intestinal crypt hypertrophy [1,2,3]. In addition to the possibility of mild enteropathy, the presence of advanced lesions characterized by intraepithelial lymphocytosis and intestinal crypt hypertrophy (Marsh stage 2), together with atrophy of the intestinal villi (Marsh stage 3) are regarded as histopathological confirmation of CD [3].

The term Potential Coeliac Disease (PCD) was first introduced in 1993 by Ferguson et al. [4]. It is observed in people with a genetic predisposition, who consume a gluten-containing diet and who possess CD-specific serum antibodies, but do not demonstrate any microscopic changes to the mucosa architecture of the small intestine (Marsh stage 0) or only display an increase in the number of intraepithelial lymphocytes in the local area (Marsh stage 1) [3]. Until 2013, this state was described as *latent coeliac disease*; however, its use was discontinued with the revision of the nomenclature of gluten-dependent diseases [1]. 

The only effective treatment for CD is a strict, lifelong gluten-free diet (GFD), which, in the vast majority of patients, leads to complete or at least partial remission of lesions in the small intestine together with any clinical symptoms, if present. The use of a gluten-free diet in people with PCD is controversial, and so far no commonly-accepted strategy exists for managing this disease [5]. 

The present article critically reviews existing literature data on the principles of gluten-free diets in patients with PCD.

## 2. Epidemiological Data 

Following more active screening for CD in general populations and in at-risk groups, the frequency of diagnosis of PCD has increased significantly in recent years. It is often diagnosed in first-degree relatives of CD patients, and in people with autoimmune comorbidities, especially dermatitis herpetiformis [6]. PCD is believed to constitute as much as every fifth diagnosis of CD [6,7,8] with a greater frequency reported by some authors in recent years [8]. However, in view of the commonly-known limitations of CD tests, these findings may be significantly overestimated. Nevertheless, compared to overt CD, PCD tends to be diagnosed in slightly younger patients [8,9] and is observed more often in women [8]. This younger age of onset may support the hypothesis that it represents an earlier phase of overt CD. 

## 3. Verification of a Diagnosis of PCD 

In any case where PCD is suspected, the diagnosis must be verified. Most importantly, it is always necessary to determine whether sufficient amounts of gluten are present in the diet, and whether gluten is being limited or completely eliminated, either consciously or unconsciously. Consuming a low-gluten diet may result in the resolution of atrophic changes in the small intestine [10]. However, in such cases, it is proposed that the small intestine should be re-biopsied after gluten challenge. As gluten sensitivity varies so much between CD patients, it is difficult to set precise guidelines regarding the duration of the challenge, or the minimum amount of gluten necessary to elicit the development of advanced lesions; however, it seems that, for most adults, it should be sufficient to consume one to three slices of gluten-based bread over a period of two to six weeks [2].

In cases of suspected PCD, genetic tests should be performed on human leukocyte antigen (HLA) genes, which have been associated with the development of CD. Although nearly 30–40% of people in the general population have at least one of the HLA molecules (HLA-DQ2.5, DQ2.2, DQ8, DQ7.5), a negative genetic test result almost completely excludes the possibility of CD and PCD. However, patients with PCD may have a slightly different genetic profile to patients who demonstrate advanced atrophic changes in the mucosa of the small intestine. PCD patients are more likely to demonstrate the HLA-DQB1 * 0302 and HLA-DQB1 * 0603 alleles, and less likely to demonstrate DQB1 * 02 homozygosity [11]. In addition, they tend to demonstrate a different distribution of six gene polymorphisms (c-REL * G, one marker of KIAA1109/IL-2/IL-21, IL-21, IL-2, KIAA1109 and c-REL) [12]. 

Serological diagnostics of CD are usually performed with anti-tissue transglutaminase (anti-TG2), anti-endomysial (EmA) and anti-deamidated gliadin peptide antibodies (anti-DGP) [3]. Both positive serum anti-TG2 and EmA are needed for a diagnosis of PCD [1,2,13]. A positive anti-DGP test result would reinforce a diagnosis of PCD; however, due to the lower value of these antibodies in CD diagnostics, they are not included in the definition of PCD [3]. 

Currently, the most commonly used, most widely available and objective CD serological marker is anti-TG2. However, while it is characterized by the highest sensitivity of all currently available serological tests (about 95%), EmA is still considered the most specific in CD diagnostics (97–100%). The expertise of the laboratory and the selection of the test kit have a great effect on the accuracy of the CD antibody test results. The accuracy of the CD antibody test is strongly dependent on the expertise of the laboratory and the choice of test. Whenever the reliability of the test or circumstances of testing are questionable, for example, in cases where initial testing is performed with a rapid antibody-detection kit or by laypeople or untrained medical staff, any positive test result should be confirmed by a laboratory-based quantitative test. Scientific societies emphasize the need for constant quality control and systematic supervision of testing laboratories at the national and international level in order to increase the validity of serological tests [3]. 

Elevated, but not high, levels of anti-TG2 can occur in many conditions other than CD, such as autoimmune diseases, including especially inflammatory bowel diseases and primary biliary cirrhosis [14], as well as Goodpasture syndrome, granulomatosis with polyangiitis (formerly called Wegener’s granulomatosis), rheumatoid arthritis, systemic lupus erythematosus, systemic sclerosis, psoriasis [15] and type 1 diabetes mellitus [16]. Elevated levels of anti-TG2 are also found in non-autoimmune diseases such as connective tissue diseases [14], non-autoimmune cirrhosis [17] and linear IgA bullous dermatosis [15]. False positive results can be obtained for EmA in cases of Down’s syndrome [18], infantile cerebral palsy [19], infectious febrile illness [20] and end-stage heart failure [21], although such results are much less common than false-positive anti-TG2. Non-CD anti-TG2 and EmA seropositivity is often transient, and may occasionally be found in healthy subjects [22,23]. Spontaneous serological negativity is particularly common in patients with type 1 diabetes [24]. It is possible that patients with false-positive specific antibodies and a normal microscopic image of the mucosa of the small intestine may be misdiagnosed with PCD. 

Patients with PCD typically demonstrate significantly lower levels of anti-TG2 than those with atrophic changes [7,25,26]. A small intestine biopsy is always required to establish the diagnosis of PCD. Pediatric patients with PCD do not meet the no-biopsy strategy criteria, as they rarely demonstrate high serum anti-TG2 concentration ≥ ten times the upper limit of the normal range [3]. 

Another key element in the correct diagnosis of PCD is reliable histopathological evaluation. As inflammatory changes are commonly found in foci in the small intestine (“patchiness”) and are restricted to the duodenal bulb (ultrashort coeliac disease), it has long been recommended to collect at least four [2] or five [3] biopsies of the small intestine mucosa, including at least one from the duodenal bulb. Taking fewer than four biopsies may result in a false negative result [27]. To avoid diagnostic errors, and to avoid overlooking atrophic changes in a patient with serological CD markers, it is important to follow the correct methodology when collecting small intestine biopsies: the tissue material should be spatially oriented on cellulose paper (orientation) and be fixed, stained and interpreted by an experienced histopathologist. It is recommended that immunohistochemical staining protocols intended for histopathological diagnosis should be based on anti-CD3 monoclonal antibodies [2,28]. 

In addition, taking into account the large high interobserver variability associated with such protocols [29], many authors suggest that in seropositive patients without typical atrophic changes, histopathological assessments should be revised by another expert, especially when the first assessment was performed in a non-specialist center [5,30]. Some rare variants of CD also exist, whose inflammatory lesions may be located beyond the reach of classic gastroduodenoscopy. Patients with such variants may benefit from a capsule endoscopy examination of the small intestine [31].

Diagnosis is also complicated by the low specificity of benign inflammatory lesions, i.e., lymphocytic duodenosis, which are classified as Marsh type 1. These lesions are also characterised by an increase in the number of intraepithelial lymphocytes to above 25 lymphocytes per 100 enterocytes in structurally-correct small intestine mucosa. In the general population, lymphocytic duodenitis may affect 5.4% of the population [32], with lymphocytic duodenosis being identified in a range of infectious diseases of the gastrointestinal tract, especially autoimmune atrophic gastritis [33], *Helicobacter pylori*-related gastritis, AIDS enteropathy, Whipple’s disease, small intestinal bacterial overgrowth and post-viral enteropathy [32], as well as hypersensitivity to milk, soy, fish, eggs or other nutrients [2]. 

The numbers of intraepithelial lymphocytes in the mucosa of the small intestine are also often elevated in intestinal disorders such as autoimmune enteropathy and Crohn’s disease, as well as in extraintestinal autoimmune disorders, such as autoimmune thyroiditis, type 1 diabetes, rheumatoid arthritis, multiple sclerosis, systemic lupus erythematosus and systemic sclerosis. Establishing a correct diagnosis is further complicated by the fact that patients with these diseases often have serological tests for CD, due to their common co-occurrence with CD, and these serological tests are more likely to return false positives [32]. The co-occurrence of mild enteropathy with elevated intraepithelial lymphocyte number may also be associated with pharmacotherapy with such agents as proton pump inhibitors, methotrexate, azathioprin, nonsteroidal anti-inflammatory drugs, olmesartan or ipilimumab [32].

## 4. Histological Features

Despite there being a lack of severe damage to the mucosa of the small intestine, PCD patients may often experience inflammatory lesions, which can be detected by immunohistochemistry. It has been found that 70.8% of PCD patients demonstrate increased numbers of CD25 + intraepithelial lymphocytes in the lamina propria, as well as increased expression of ICAM-1 and crypt HLA-DR (crypt HLA-DR), indicating the activation of immune processes in the epithelium, lamina propria and intestinal crypts [34]. Patients with CD, including PCD, also have a significantly higher density of γδ T-cell receptor-bearing intraepithelial lymphocytes (CD3TCRγδ IEL); although this density is not a pathognomonic feature of CD, it probably heralds the development of advanced inflammatory lesions in PCD patients [35].

In addition, in both PCD and overt CD, anti-TG2 deposits are often found in the epithelium of the small intestine and in the perivascular area. The prevailing opinion is that their presence in PCD patients is a strong predictor of the development of atrophic lesions of the small intestine, and their presence in the circulation has been attributed to their release from the intestinal mucosa (“spillover”) [13,36]. In patients with histopathologically confirmed CD and PCD, testing based on intestinal anti-TG2 deposits is estimated to have a sensitivity of 100% and a specificity of 99% [37]. Similar to overt CD, the serum profile and metabonomic signature of PCD [38], and its associated intestinal inflammatory changes [34,35,36], suggest that PCD may represent an early stage of CD, and not a separate disease entity. The progression of the gluten-induced immune response is reflected in the expression of interleukin 12 (IL-12). While IL-12 has been found to mediate the immune response in CD, its expression in the mucosa of the small intestine is suppressed in PCD, which suggests that the mechanisms of cellular damage present in overt CD are not activated [12]. 

## 5. Clinical Picture

PCD may be asymptomatic, or it can manifest as gastrointestinal or parenteral-related clinical symptoms which range from mild to severe. It is most often diagnosed during serological screening in risk groups, i.e., first-degree relatives of CD patients, patients with type 1 diabetes and those with autoimmune thyroiditis. Unlike the adult population, most children with PCD are asymptomatic [9,39]. Symptomatic patients, especially adults, are more likely to present gastrointestinal symptoms such as intestinal malabsorption, chronic diarrhea and recurrent abdominal pain, but less likely to demonstrate parenteral symptoms such as anemia, hypertransaminasemia, osteopenia, stomatitis, recurrent miscarriage or shortness of stature [8].

## 6. Prognostic Markers

Factors are being sought which would allow for the identification of patients with a high probability of developing overt CD. Over longer follow-up periods, one predictor of atrophic changes may be repeated seropositivity in the range of celiac-specific antibodies [40], as well as their initial higher concentration [41]. Auricchio et al. [39] propose the following factors as good predictors in pediatric patients: older age (<3 years vs. ≥3 years), HLA DQB1 * 02 homozygosity, presence of endothelial lymphocytosis (Marsh 1 vs. 0), higher number of γδ lymphocytes in the initial small intestine biopsy (11.9 vs. 6.44) and the presence of anti-TG2 deposits in the mucosa of the small intestine. In addition, the presence of intestinal autoantibody deposits demonstrates high sensitivity and specificity in prognosing subsequent coeliac disease (both features: 93%) [41]: more so than the number of CD3 + lymphocytes, the density of γδ-lymphocytes and the presence of endothelial lymphocytes at the tips of the intestinal villi. However, neither elevated intraepithelial lymphocyte count nor an increase in anti-TG2 deposits were found to predict the development of active histopathological changes [8]. 

In both active CD and PCD patients, it has been proposed that anti-TG2 detection is more efficient when performed on supernatant taken from 24 h culture of small intestine cell biopsies [27]. Auricchio et al. [42] also propose that the presence of the *IL2/IL21* gene marker on chromosome 4 is a good predictor of the development of advanced histological changes. However, the significance of factors predicting the progression of PCD to villus atrophy, such as the coexistence of other autoimmune diseases and gender, remains unclear [39]. Most of the parameters that help identify patients with PCD who go on to develop atrophic changes in the small intestine mucosa are not routinely measured; these patients should be monitored in tertiary care centers with access to these studies. 

## 7. Natural Course 

The natural course of PCD is difficult to determine, especially as many patients switch to a gluten-free diet (GFD) immediately after diagnosis, either of their own volition or in accordance with medical recommendations. Studies on this subject are scarce, and most have been performed on small groups of patients (Table 1). However, monitoring studies confirm that only a small proportion of patients with PCD will develop the histopathological changes typical for overt CD. Earlier studies on small groups of pediatric [26] and adult [43] patients suggest that most PCD patients may be subject to the development of atrophic lesions; however, the recently-published results of a long-term observation of large groups of children on a gluten-containing diet over nine years indicate that intestinal villus atrophy developed in only about 1/3 of them [42]. Therefore, even symptomatic patients consuming gluten for many years may not display any histopathological changes and may even demonstrate any spontaneous resolution of clinical symptoms [6]. The few studies based on an observation period of at least several years show that microscopic changes in the small intestine may develop at any time, and not always during the first two years of follow-up [39].

A key element influencing the natural course of PCD is the clinical picture of the disease. In the majority of symptomatic patients, the introduction of a GFD results in clinical [6,8,9,25,26,43] and serological remission [8,26,43]. Data on the regression of microscopic changes, if they were initially present, are sparse and inconsistent [9,43]. Symptomatic patients on a diet containing gluten mostly remain symptomatic [43] and seropositive [43,44], and histopathological changes typically increase [26,44]; however, in one study, data suggest that microscopic changes were not found to progress in up to 80% of patients at one-year follow-up [44]. 

Among asymptomatic patients on a diet containing gluten, serological tests remain positive in 43–62.5% [8,25,42] and negative in 14.6–86% [7,8,25,40,42], with values fluctuating in 4–37% [7,8,25,40,42]. A small proportion of asymptomatic patients (5–35.7%) were found to develop atrophic changes of the small intestine typical of CD during a two- to nine-year follow-up [6,7,25,40].

## 8. Treatment Principles

Decisions about the management of patients with PCD are difficult. On the one hand, there is reliable evidence that after the elimination of gluten from the diet, a significant proportion of patients experience clinical improvement or remission, for symptoms both inside and outside the gastrointestinal tract. A delay in diagnosis, and thus treatment of celiac disease, is also associated with a reduction in the quality of life, greater school and work absenteeism, more frequent use of medication and the use of healthcare facilities, and perhaps also an increased risk of developing neoplastic diseases, especially lymphoproliferative diseases. In addition, some complications, such as growth failure in children, osteoporosis and tooth enamel defects, may be irreversible if left untreated [5]. On the other hand, a few PCD patients may experience spontaneous resolution of clinical symptoms and serological negativity despite continued gluten consumption; in such cases, it would be difficult to justify the use of a GFD, and its associated financial burdens and social constraints. Moreover, large population studies of adults without CD in America have confirmed an inverse relationship between gluten consumption and the risk of developing type 2 diabetes [45], and it also appears that avoiding gluten may increase the risk of cardiovascular complications due to lowered whole grain consumption [46]. Moreover, it has been shown that non-CD patients avoiding gluten consumption have a higher risk of developing inflammatory bowel diseases, irritable bowel syndrome, thyroid diseases, lupus and autism spectrum disorders [47].

**Figure 1:**
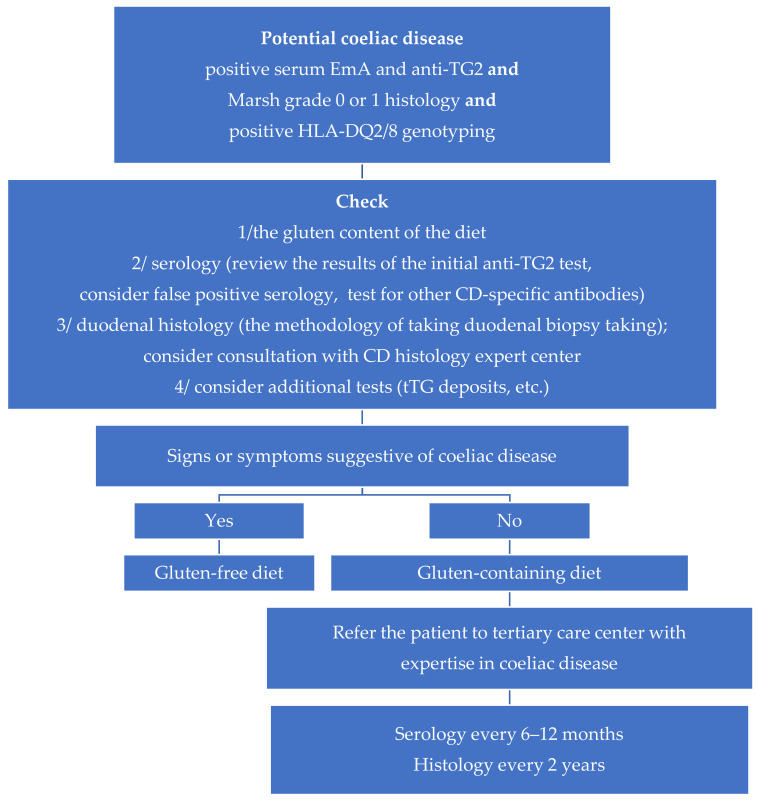
A proposed rational approach to treating patients with potential coeliac disease.

There are no generally accepted principles for the therapeutic management of PCD. Given the clinical benefits for most symptomatic patients, the prevailing view in the literature is to include GFD in their treatment. This applies to patients with gastroenterological symptoms such as diarrhea, abdominal pain and constipation, as well as parenteral symptoms such as anemia, osteoporosis or migraines [13,48]. In the case of asymptomatic patients, it is suggested that they remain on a gluten-containing diet, but under close medical supervision. So far, no uniform, generally-accepted principles have been defined for monitoring PCD patients consuming a general diet [7,9,25,30,40]. In most expert centers, patients are monitored clinically and serologically every six to twelve months [2,8], and histopathologically every two years [8]. A proposed scheme of therapeutic management in patients with PCD is given in Figure 1. 

## Figures and Tables

**Table 1:** Results of available evidence of a different dietary approach for potential coeliac disease.

First Author and Publication Date	Study Population	Results	Limitations
	**Pediatric Studies**		
Paparo F. 2005 [34]	18/24 children with symptoms suggestive of CD or belonging to “at-risk” groups on a gluten-containing diet	antibody negativization in 3/18, villous atrophy development in 2/6 who underwent a second biopsy	small sample size
	6/24 children with symptoms suggestive of CD or belonging to “at-risk” groups on GFD	all demonstrated positive clinical and serological response	
Kurppa K. 2010 [26]	8/13 children with signs/conditions suggestive of CD on a GFD	villous atrophy in 5/8 after the first year and in next 2/8 after the second year	only four distal duodenal biopsies taken, short follow-up period
	5/13 children with signs/conditions suggestive of CD on a GFD	positive clinical and serological response in all after a year	
Tosco A. 2011 [25]	86 asymptomatic children on a gluten-containing diet	persistent positive serology in 52.9%, completely or persistently negative serology in 14.6%, fluctuation of antibody titers in 32.6%; villous atrophy in 12/39 (30.8%) who underwent a repeat biopsy within 3 years	only four distal duodenal biopsies taken
	20 children with persistent symptoms/conditions suggestive of CD put on a GFD	no clinical response in 9/20	
Lionetti E. 2012 [7]	21 asymptomatic children left on a gluten-containing diet for two years	negative serology in 18/21 (86%), fluctuating antibody level in 2/21 (9%), histologically confirmed CD in 1/21 (5%)	
Auricchio R. 2014 [42]	175 asymptomatic children on a gluten-containing diet	persistently elevated anti-TG2 level in 43%, negative anti-tTG in 20% and fluctuant anti-TG2 with transiently negative values in 37% during follow-up, normal duodenal architecture at 3, 6 and 9 years of follow-up in 86%, 73% and 67% patients, respectively	
Mandile R. 2018 [9]	35 symptomatic children placed on GFD	positive clinical response in 19/35 (54%), partial clinical response in 2/35 (6%), no clinical response in 14/35 (40%), no significant differences in terms of Marsh grade, lamina propria *CD25+* cells, CD3+, γδ+ intraepithelial lymphocytes density and intestinal anti-TG2 deposits after at least 1 year on GFD	
Lionetti E. 2019 [40]	23 asymptomatic children on gluten-containing diet	negative serology up to 10 years of follow-up from the first biopsy in 19/23 (83%), fluctuating antibody values and persistently negative biopsy in 1/23 (4%), overt CD development in 3/23 (13%)	
Auricchio R. 2019 [39]	280 children with symptoms, familiar risk or autoimmune comorbidity followed on a gluten-containing diet over a median follow-up of 60 months	a GFD introduction (without biopsy) for symptoms developed during the follow-up in 39/280 (13.9%); a flat mucosa development in 42/280 (15%); negativization of anti-TG2 or EMA in 89/280 (32%), 166/280 (59.2%) remained potential at 12 years of follow-up	
	**Adult Studies**		
Kurppa K. 2009 [43]	10/23 adults with signs suggestive of CD on a gluten-containing diet	villous height/crypt depth ratio decreased, intraepithelial lymphocytosis and serum endomysial antibody titers remained increased in all; the symptoms persisted in all	Marsh II included in study population
	13/23 adults with signs suggestive of CD on a GFD	villous height/crypt depth ratio increased, intraepithelial lymphocytosis decreased, serum endomysial antibody titers normalized, the symptoms alleviated in all	
Biagi F. 2013 [6]	24 adult patients’ symptoms of malabsorption, associated diseases or familiarity for CD maintaining a GFD	flat mucosa development in 5/14 within 12 ± 8 months; preserved mucosal architecture in 9/14 within 30 ± 29 months, spontaneous clinical remission in 3/10 without subsequent biopsy	retrospective study
	23 adult patients with symptoms of malabsorption or associated diseases put on a GFD	clinical improvement in all (19/23) with gastrointestinal symptoms or dermatitis herpetiformis	
Volta U. 2016 [8]	16 asymptomatic adult patients left on a gluten-containing diet over a median follow-up of 3 years	diarrhea/anemia and subtotal villous atrophy development in 1/16 (6%), EmA/anti-TG2 disappearance in 4/16 (25%), antibody fluctuation in 1/16 (6.3%), antibody persistence in 10/16 (62.5%), no histologic changes in 10 patients with persistent or fluctuating antibody positivity	small sample size
	61 adult symptomatic patients put on a GFD over a median follow-up of 3 years	all demonstrated significant clinical improvement and negativization of antibodies	
	**Mixed-Age Studies**		
Kondala R. 2016 [44]	57 patients (children and adults) with IBS-like symptoms, iron-deficiency anemia or familiarity for CD on gluten-containing diet followed up for 12 months	serological negativization in 12/57 (21.1%), non-progressive duodenal histology in 46/57 (80%), histological worsening from Marsh-0-II to Marsh III in 4/57 (7%)	Marsh 2 included in the study population, short-term follow-up

CD–coeliac disease; GFD–gluten-free diet; Anti-TG2–anti-tissue transglutaminase antibodies; EmA–endomysial antibodies; IBS-irritable bowel syndrome.
